# Accumulation of Anthocyanidins Determines Leaf Color of Liquidambar Formosana as Revealed by Transcriptome Sequencing and Metabolism Analysis

**DOI:** 10.3390/cimb44010018

**Published:** 2022-01-07

**Authors:** Jiuxin Lai, Furong Lin, Ping Huang, Yongqi Zheng

**Affiliations:** 1 State Key Laboratory of Tree Genetics and Breeding, Key Laboratory of Tree Breeding and Cultivation of the State Forestry Administration, Research Institute of Forestry, Chinese Academy of Forestry, Beijing 100091, China; jackolai@126.com (J.L.); Linfurong@caf.ac.cn (F.L.); Huangping@caf.ac.com (P.H.); 2 Turfgrass Research Institute, College of Forestry, Beijing Forestry University, Beijing 100083, China

**Keywords:** *Liquidambar formosana*, leaf coloration, flavonoid, transcriptome analysis, anthocyanidin biosynthesis pathway

## Abstract

*Liquidambar formosana* is important for its ornamental value in China; it is increasingly used for landscaping and gardening trees due to its diverse leaf colors and seasonal changes. Varieties including either a fixed leaf color, the purplish ‘Fuluzifeng’ (ZF), or seasonal changes in leaf color, the reddish ‘Nanlinhong’ (NLH) have been bred and registered as new plant varieties under the International Union for the Protection of New Plant Varieties (UPOV) system. To gain practical insights into the anthocyanin biosynthetic process, transcriptome sequencing (Illumina) was performed to clarify the metabolic pathways present in the three seasonal changes in leaf colors in NLH and in the springtime purple-red color of ZF. qRT-PCR was used to verify the speculation. Based on the differentially expressed genes and flavonoids analyses, the spring, summer, and autumn leaves of NLH were compared to study the seasonal differences. NLH and ZF were compared to study the formation mechanism of the two leaf colors in spring. Transcriptome sequencing produced a total of 121,216 unigenes from all samples, where 48 unigenes were differentially expressed and associated with the anthocyanidin pathway. The expression levels of *LfDFR* and *LfANS* genes corresponded to the accumulation of concentrations of cyanidins in spring (NLHC) and autumn leaves (NLHQ), respectively, with different shades of red. Moreover, the *LfF3′5′H* gene corresponded to the accumulation of flavonols and delphinidins in purple-red leaves (ZFC). Cyanidin and peonidin were the key pigments in red and dark-red leaves, and purple-red leaves were co-pigmented by cyanidins, pelargonidins, and delphinidins.

## 1. Introduction

*Liquidambar formosana* Hance is a deciduous ornamental tree species and is widely distributed in Southeast Asia [1,2]. Its wood is widely used for flake and strand boards. The species is increasingly being used in landscaping and gardening in subtropical regions due to its diverse leaf colors and seasonal changes, especially in autumn. Varieties including either a fixed leaf color, the purplish ‘Fuluzifeng’ (ZF), or seasonal changes in leaf color, the reddish ‘Nanlinhong’ (NLH), have been developed and registered as new plant varieties under the UPOV system. The variety ZF retains a purple-red leaf color throughout the growing season of a year, while the variety NLH has red leaves in spring that turn into green in the summer and red again in autumn. Leaf coloration is usually considered a consequence of anthocyanin accumulation. Archetti et al. [3] indicated that anthocyanins are not present in the leaves during spring and summer but begin producing actively toward the beginning of autumn. This study was intended to understand the biosynthetic pathway related to anthocyanin, to clarify key genes regulating the biosynthesis of the leaves in different seasons, and to try to investigate why spring leaves present red and purple-red colors.

Flavonoids have many functions in the regulation of pigmentation, plant development, and photo-protection [4]. Advances in flavonoid biosynthesis research have identified an increasing number of such functions. Assorted flavonoids are found in plants as a group of secondary metabolites [5]. Along with carotenoids and chlorophylls, flavonoids are known as the main determinants of pigmentation and are responsible for the various colors of leaves, especially for anthocyanidins. The dominant pigments of anthocyanidins in leaves are cyanidins (penodins), pelargonidins, and delphinidins (malvidins), which determine the red and blue hues [6,7,8]. The colorful pigments are the results of the number of hydroxyl groups and methyl groups [9]. However, due to anthocyanins’ structural diversity [10], the molecular mechanism regulating anthocyanin biosynthesis has not been identified in many plant species [11]. Although several studies have been conducted on the metabolism of flavonoids and anthocyanidins in ornamental plants [5,12], the molecular mechanism of leaf coloration of *L. formosana* has not been fully addressed.

The leaf coloration mechanism is complicated, as the biosynthetic pathway of anthocyanidin is affected by multiple endogenous and exogenous patterns, such as pH condition, illumination intensity, temperature variation, diseases, and insects. Anthocyanin accumulation-related genes have been studied in many model plants, such as Arabidopsis and Petunia [13]. The process of pigmentation has been found in the cytosol and involved various enzymes [9]. Previous studies suggested that the DFR and MYB113 genes accumulate during autumn, while the pigments are produced in the senescent *L. formosana* leaves [1]. It is worth discussing why the leaves turn red or purple-red in spring and how the mechanism regulates leaf color throughout the growing season. Transcriptome and metabolism datasets have been used in conjoint analyses in several plants, including tobacco [14], kale [15], and strawberry [12], which confirmed the method’s feasibility and rationality. In this study, transcriptome sequencing was performed to further elucidate the metabolic pathways of NLH leaves in different seasons and in the purple-red leaves of ZF in spring. Flavonoid composition was identified by Ultra Performance Liquid Chromatography–Electrospray Ionization–Tandem Mass Spectrometry (UPLC-ESI-MS/MS), and the RNA-seq was performed by Illumina sequencing platform to screen novel differentially expressed genes (DEGs). The networks of genes and metabolites were mapped to highlight the process of leaf color changing during spring, summer, and autumn, and the color difference between the two varieties of spring leaves.

## 2. Materials and Methods

### 2.1. Plant Materials

**Figure 1:**
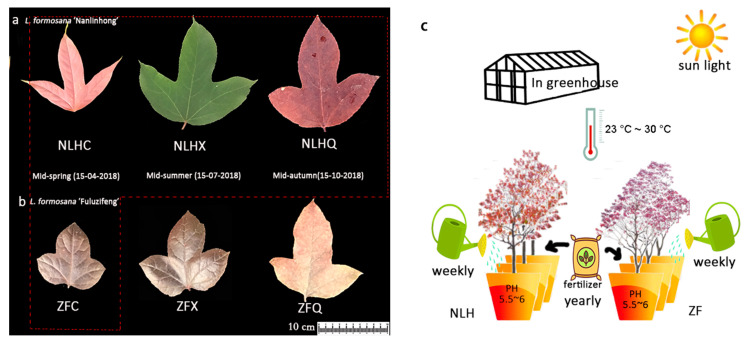
Different developmental stages of two varieties of *L. formosana*. (**a**): *L. formosana* ‘Nanlinhong’; (**b**): *L. formosana* ‘Fuluzifeng’; Four samples in the red box were used for transcriptome and metabolome detection. (**c**): Experiment design. During the growing season, 0.061 g/100 mL imidacloprid (Essence Co., Ltd., Nanjing, China) was used for pest control every three months (after sampling); plants were manually irrigated without herbicide and were watered once per week; organic fertilizer (Jing Longyuan Technology Co., ltd., Beijing, China) was only used in the fall of each year, and there was no shading or fill-up.

Leaf samples were collected from three-year-old trees of the two grafted varieties from the greenhouse of the Chinese Academy of Forestry (Beijing, China) under natural light with day (highest) and night (lowest) conditions, with temperatures from 30 °C to 23 °C, and with soil pH condition around 5.5~6. The three seasons with different leaf colors of NLH (red leaves in spring, NLHC; green in summer, NLHX; dark-red in autumn, NLHQ, Figure 1a) were selected for metabolism and transcriptome analysis, as well as the purple-red leaf of ZF (ZFC, Figure 1b). The fresh leaves were collected at 10:00 a.m. and kept in liquid nitrogen, then stored at −80 °C before further treatment. The samples of NLHC, NLHX, and NLHQ were used to construct twelve libraries with three replicates from three drafted individuals. Three stages of two varieties were all used for qRT-PCR analysis.

### 2.2. Detection of Flavonoids in Leaves

Metware Biotechnology Co., Ltd. (Wuhan, China) was used to perform multiple-reaction monitoring (MRM). Each sample was pulverized and finely ground for 2.5 min at 25 Hz in a mixer mill (MM 400, Retsch, Haan, Germany) with triplicate injections. Then, 1.0 mL 70% aqueous methanol was used to extract the obtained powder with 100 mg at 4 °C. The extracts were centrifuged at 10,000× *g* for 10 min, then absorbed in 250 mg/3 mL CNWBOND Carbon-GCB SPE Cartridge (HYSBEQ-CA1663, ANPEL, Shanghai, China) and filtered using a Nylon Syringe Filter with 13 mm* 0.22 µm pore size (SCAA-104, ANPEL, Shanghai, China) prior to LC-MS analysis [16]. Filtered extracts were processed using Ultra Performance Liquid Chromatography (UPLC, Shim-pack UFLC SHIMADZU CBM30A system, Shimadzu, Kyoto, Japan) and Electrospray Ionization–Tandem Mass Spectrometry (MS, Applied Biosystems 6500 Q-TRAP, Thermo Fisher, Boston, MA, USA). The analytical conditions were as follows [16,17]:

                      UPLC column: Waters ACQUITY UPLC HSS T3 C_18_, pore size 1.8 µm, 2.1 mm × 100 mm (Milford, MA, USA);

    Solvent system: Mobile phase A (0.04% acetic acid in water);                          Mobile phase B (0.04% acetic acid in acetonitrile);

    Gradient program (A:B): 95:5 *v/v* at 0 min;                                                5:95 *v/v* at 11.0 min;                                               5:95 *v/v* at 12.0 min;                                               95:5 *v/v* at 12.1 min;

       Flow rate: 0.40 mL/min;

      Injection volume: 2 µL;

Temperature: 40 °C.

The analysis was carried out in the ESI-MS-MS ion mode. After UPLC, an electrospray ionization triple quadrupole-linear ion trap was alternatively connected to detect the effluent. Linear ion trap (LIT) and triple quadrupole (QQQ) scans were acquired on a triple quadrupole-linear ion trap mass spectrometer, API 6500 Q-TRAP LC/MS/MS System equipped with electrospray ionization (ESI) Turbo Ion-Spray interface, operating in a positive ion mode and controlled by the Analyst 1.6.3 software (AB Sciex, Waltham, MA, USA). The MRM for each variety was performed in triplicate. The ESI source operation parameters were as follows [16,17]:

            Electrospray ionization (ESI) temperature: 500 °C;

   Ion spray voltage (IS): 5500 V;

Curtain gas, CUR: 25.0 psi;

          Collision-activated dissociation (CAD) gas: high;

            Declustering potential (DP): specific optimization;

        Collision energy (CE): specific optimization.

### 2.3. RNA Extraction and Sequencing

The total RNA was extracted using a modified CTAB method with RNase-free DNase I (RR047Q, Takara, Beijing, China) treatment. The quantity of extractions was characterized on a 1% agarose gel and determined by Kaiao K5500 spectrophotometer (Kaiao, Beijing, China) with Agilent 2100 RNA Nano 6000 Assay Kit (Agilent, CA, USA), OD260/280 > 2.1 and RIN number of >7.0. All qualified samples were sent to the Anoroad genome Corporation (Beijing, China) to construct the twelve libraries and then sequenced by Illumina Hiseq 4000 platform (Illumina, San Diego, CA, USA). In brief, mRNA with Oligo (dT)-attached magnetic beads were isolated by the high-quality extractions. A fragment buffer was added to the mRNAs to break them into short fragments. Random hexamer primers were used to synthesize first-strand cDNA with the templates of short fragments. Then, d-NTPs, RHase-H, DNA-polymerase-I, and buffer were added to synthesize the second-strand. Fragments of cDNA were purified by QIAQuick Kit with EB buffer (Mbbiotech, Guangzhou, China). The purified products were used for end-repairing with the addition of poly A and sequencing adapters. The cDNA libraries were amplified by PCR with recycled fragments on 2% agarose gel.

### 2.4. De Novo Transcriptome Assembly, Unigene Annotation, and Differential Expression Analysis

Low-quality reads contained adapters, and unknown bases were removed to filter the raw pair-end (150 bp) reads. The FastQC (Babraham Institute, Cambridge, UK) was used to verify the sequence quality, including GC content, Q20, and Q30 of the clean data. The clean data of all the twelve samples were combined for reference-free de novo assembly using Trinity Release 2.4.0 (Hell’s Kitchen, Manhattan, NY, USA) and deposited in NCBI Genebank with accession number (GILF01000000) [18,19]. Trinity grouped transcripts were used to build a De Bruijn diagram and referred to as a “gene” based on shared sequence content. Uni-transcript sequences were compared by BLAST (E-value < 0.00001) [20] against the Nr, SwissProt, GO, eggNOG, and KEGG databases. Unigene annotation information was determined by Trinotate Release v3.0.2 (https://github.com/Trinotate, accessed on 25 December 2021). Alignment results of each sample were compared by Bowtie [21] and estimated expression level by RNA-seq utilizing expectation maximization (RSEM) [22], three biological replicates in each case. Reads Per Kilobase Million Mapped Reads (RPKM) were used to distinguish the different unigene expression levels among samples. Then, differentially expressed unigenes (DEGs) were screened by DESeq via pairwise comparisons with false discovery rate (FDR < 0.05) and |log2 fold change (FC)| ≥ 1 using R package edgeR [23].

In order to validate RNA-seq results and the gene functions related to anthocyanin pathway, Roche Light Cycler 480 machine (Roche, San Francisco, CA, USA) was used to perform Real-time-quantitative PCR (qRT-PCR) on a 96 real-time system with SYBR^®^ Premix Ex TaqTM (RR903a, TaKaRa, Beijing, China). Primer 5.0 was used to design the primers (Supplementary Table S7). Synthesis of cDNA and qRT-PCR was performed using previously described methods with triplications of each sample [24], and the inference gene β-actin [25] was used to normalize gene expression levels.

## 3. Results

### 3.1. Major Flavonoid Compounds in the Differently Colored Leaves of L. formosana

**Figure 2:**
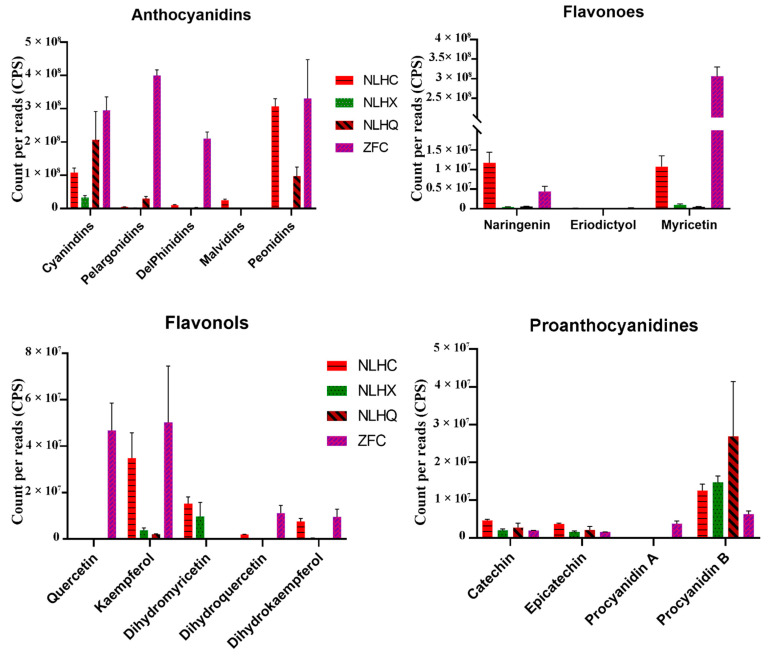
Flavonoid compositions of in three season leaves of *L. formosana* ‘Nanlinhong’ (NLH) and spring of *L. formosana* ‘Fuluzifeng’ (ZF). NLHC refers to the spring leaves of NLH with red color, NLHX refers to the summer leaves of NLH with green color, NLHQ refers to the summer leaves of NLH with dark red color, and ZFC refers to the spring leaves of ZF with purple color.

Fifteen types of anthocyanins were found in the two varieties of *L. formosana,* divided into five anthocyanin groups, namely cyanidins, peonidins, pelargonidins, delphinidins, and malvidins (Supplementary Table S1). Cyanidins and peonidins were the main compounds of the detected flavonoids in the leaves of NLH in the three seasons (Supplementary Table S1). More than 50% of the detected compounds were anthocyanins and exist in the summer leaves of NLH. The anthocyanin concentration was nearly ten times lower in the summer than in spring and autumn. There were significantly different compositions found in the various seasons of NLH (Figure 2). Cyanidin was only found in the red spring leaves (NLHC), as was delphine chloride. Cyanidin 3-O-glucoside was only found in the autumn dark red leaves (NLHQ) (Supplementary Table S1). Cyanidins showed the highest concentrations in the dark red leaves (NLHQ), with twice higher levels than red leaves (NLHC) and six times levels higher than the green leaves (NLHX). There were also significant differences in the composition and concentration of anthocyanins. Cyanidin O-diacetyl-hexoside-O-glyceric acid and delphinidin 3-O-rutinoside were two of the unique compounds in ZFC (Supplementary Table S1). Pelargonidins and delphinidins have more actively accumulated in ZFC. In spring, the variety ZF showed a higher accumulation level of anthocyanidins than the variety NLH. Myricetin was found significantly in ZFC. However, the concentration of procyanidins was higher in the NLH variety.

### 3.2. Sequence Assembly and Gene Annotation

Twelve cDNA libraries of NLHs (spring, summer, and autumn leaves) and ZF (spring leaf) were sequenced and constructed, resulting in an amount of 83.02 GB clean reads (Supplementary Table S2). The Q30 was greater than 93.8% in each sample, corresponding to base reads with low-quality rates below 1%. The GC contents amounted to ~40.0% of the clean reads. De novo assembly of these transcriptomes resulted in 121,216 unigenes with a mean length of 782.8 bp (Supplementary Table S3). The assembly data showed a high quality of RNA sequencing, which could be used for further study. RPKM values were normalized by Pearson’s correlation matrix (Supplementary Figure S1). The clustering result showed the reliability of sample replications with identical expression profiles (R > 0.8).

**Figure 3:**
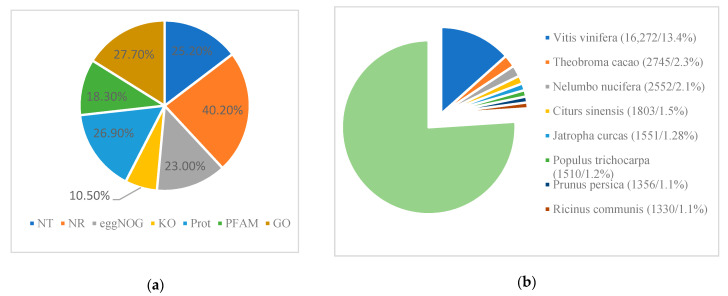
Unigene mapping. (**a**) Different database mapping results; (**b**) species matching based on the Nr database.

Most parts of the annotated unigenes (48,749, 40.2%) were assigned to the Nr protein database, while the smallest was related to the KO database (10.5%, Figure 3a). The similar genetic species were compared based on Nr database (Figure 3b). Data showed that *Vitis vinifera* (13. 4%) had the highest ranking, *Theobroma cacao* (2.3%) was the second, and then was Nelumbo nucifera (2.1%), while only 25 unigenes were assigned to existing fragment data of *Liquidambar formosana* (0.021%) and 13 unigenes to *Liquidambar styraciflua* (0.011%). The annotated unigenes were mapped to the Gene Ontology (GO) in the biological process, and 52.54% of them were related to the metabolic process. However, only 0.08% of unigenes (97 unigenes) were assigned to pigmentation function.

### 3.3. Differentially Expressed Genes and Relation with the Different Leaf Colors

We compared the read counts for each gene in leaves with different colors (based on fold change ≥ 2, and false discovery rate ≤ 0.01) to identify the differential expression genes (DEGs). A total of 3563 DEGs were found between two varieties (NLHC and ZFC) and 15,709 DEGs among three seasons in NLH (Supplementary Table S4). However, only 15 DEGs were found in the NLHC vs. NLHX vs. NLHQ (Supplementary Figure S2a). A unique gene (TRINITY_DN25298_c0_g2) was differentially expressed among all four samples (Supplementary Figure S2b). The greatest number, 13,405 DEGs, were found in the NLHX vs. NLHQ, among which 7151 genes were down-regulated, and 6396 genes were up-regulated. There were 8442 DEGs between NLHC and NLHX, with 4548 up-regulated and 3894 down-regulated. However, only 87 DEGs were found between NLHC and NLHQ, whose leaf color looked similar, and 24 genes were up-regulated, and 55 genes were down-regulated (Supplementary Figure S2c).

**Figure 4:**
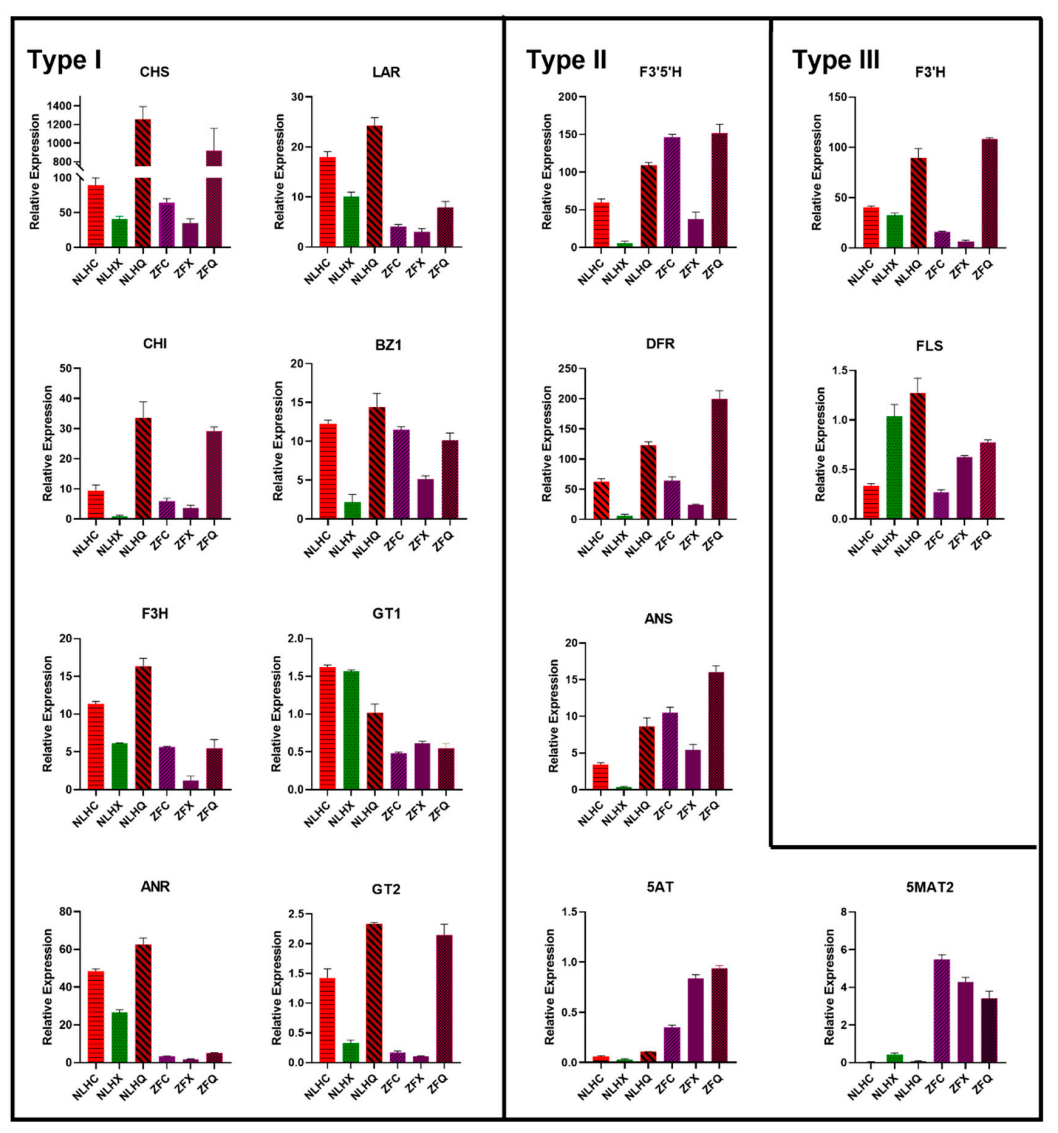
Expression levels of anthocyanin-related genes in *L. formosana* with different leaf colors. Each sample was tested in triplicate in qRT-PCR reactions, and vertical bars indicate standard errors.

The unigenes were mapped against the authoritative reference, the Kyoto Encyclopedia of Genes and Genomes (KEGG) pathways, to determine which were related to red or purple-red leaf colors. A total of 4333 DEGs were mapped KEGG pathways are shown in Supplementary Figure S3. The results revealed that 48 DEGs were assigned to the flavonoid and anthocyanin biosynthesis pathway (Supplementary Table S5). While 12 DEGs were found in NLHC vs. NLHX and 33 DEGs were found in NLHX vs. NLHQ, no DEGs were found between NLHC and NLHQ. Meanwhile, eight DEGs were shown to play key roles between two varieties in spring (NLHC vs. ZFC) (Figure 4 and Supplementary Figure S4). One DEG encoding chalcone synthase (CHS, K00660) was annotated in the flavonoid biosynthesis, but no significant difference was found between two varieties in spring. Two DEGs encode chalcone isomerase (CHI, K01859), five DEGs encode flavanone 3-hydroxylase (F3H, K00475), five DEGs encode flavonoid 3′hydroxylase (F3′H, K05280), and three DEGs encoding flavonoid 3′,5′-hydroxylase (F3′5′H, K13083). In addition, four DEGs encode flavonol synthase (FLS, K05278), six DEGs encode anthocyanidin reductase (ANR, K08695), and two DEGs encode leucoanthocyanidin reductase (LAR, K13081). In the upstream of anthocyanin biosynthesis, four DEGs encode dihydroflavonol 4-reductase (DFR, K13082), and one DEG encodes anthocyanidin synthase (ANS, K05277). Eight DEGs encode anthocyanidin 3-O-glucosyltransferase (BZ1, K12930). Several DEGs associated with other enzymes in the GT family were identified, such as one DEG for anthocyanidin 3-O-glucosyltransferase 1 (GT1, K12938), and three DEGs for anthocyanidin 3-O-glucosyltransferase 2 (GT2). Furthermore, in anthocyanidin modification, one DEG encodes Anthocyanin 5-aromatic acyltransferase (5AT, K12936), and two DEGs encode anthocyanidin 5-O-glucoside-6″-O-malonyltransferase (5MAT2, K12934).

Fifteen genes implicated in leaf coloration were used for qRT-PCR analysis, which revealed that the expression patterns were generally consistent with RNA-seq expression level (Supplementary Figure S5a) with a high correlation coefficient (R^2^ = 0.877, Supplementary Figure S5b). The expression levels of 15 genes were detected in two varieties with three seasons, namely NLHC (red), NLHX (green), NLHQ (dark red), ZFC (purple-red), ZFX (purple-red), and ZFQ (light purple-red). According to the qRT-PCR results, those gene expression levels can be separated into three groups (Figure 4). The type I included *LfCHS*, *LfF3H*, *LfCHI*, *LfANR*, *LfLAR*, *LfBZ1*, *LfGT1*, and *LfGT2*. Their expression levels in the NLHs’ leaves were higher than in the ZFs’, and most of them showed the lowest expression level in summer leaves, except for *LfGT1*. The second type included the *LfF3′5′H*, *LfDFR*, *LfANS*, *Lf5AT*, and *Lf5MAT2* genes. Their expression levels in the ZFs were higher than others in the NLHs. The expression level of *LfCHI* and *LfDFR* had significant seasonal variations. Both varieties showed the highest expression level in autumn and the lowest in summer. Type III included the *LfF3′H* and *LfFLS*. Their expressions were inconsistent in different leaves colors; for example, the *LfF3′H* genes were the highest in ZFQ but lowest in ZFX, whereas *LfBZ1*showed the highest expressed in NLHQ, and the lowest in NLHX.

### 3.4. Differential Expressed Genes and Relation with the Different Leaf Colors

**Figure 5:**
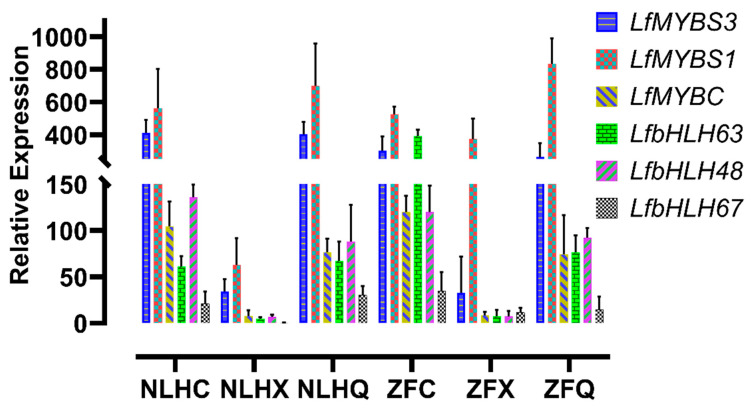
The relative expression level of TF genes on different leaf stages of two varieties (*LfMYBS3*, *LfMYBC*, *LfMYB4*, *LfbHLH48*, *LfbHLH63*, and *LfbHLH67*). Each sample was analyzed in triplicate on qRT-PCR reactions, and vertical bars indicate standard errors. NLHC refers to the spring leaves of NLH with red color, NLHX refers to the summer leaves of NLH with green color, NLHQ refers to the autumn leaves of NLH with dark red color, ZFC refers to the spring leaves of ZF with purple color, ZFX refers to the summer leaves of ZF with purple color, and ZFQ refers to the autumn leaves of ZF with purple color.

A total of 107 transcription factors (TFs) belonging to seven TF families were differentially expressed between NLHC and NLHX, 146 TF genes were found in NLHX vs. NLHQ, and none of the differential expression TF genes were found in the NLHC vs. NLHQ. Three TF genes in the NLHC vs. ZFC were MYB genes. The heatmap was used to screen the key TFs for anthocyanin synthesis (Supplementary Figure S6), and 172 differentially expressed TFs belonging to seven TF families were found in the NLHC vs. NLHX vs. NLHQ, such as MYB (45), bHLH (26), WRKY (26), AFR (13), NAC (33), SPL (19), and TCP (10) (Supplementary Table S6). Expression levels of those TFs were used to select for further analysis. Six TFs (*LfMYBS3*, *LfMYBC*, *LfMYB4*, *LfbHLH48*, *LfbHLH63*, and *LfbHLH67*) were selected for qRT-PCR analysis, which was predominantly expressed in NLHC. They showed a higher expression level in red leaves than green leaves (Figure 5).

### 3.5. Candidate Genes Responsible for the Accumulation of Anthocyanins in the Leaves of NLH

**Figure 6:**
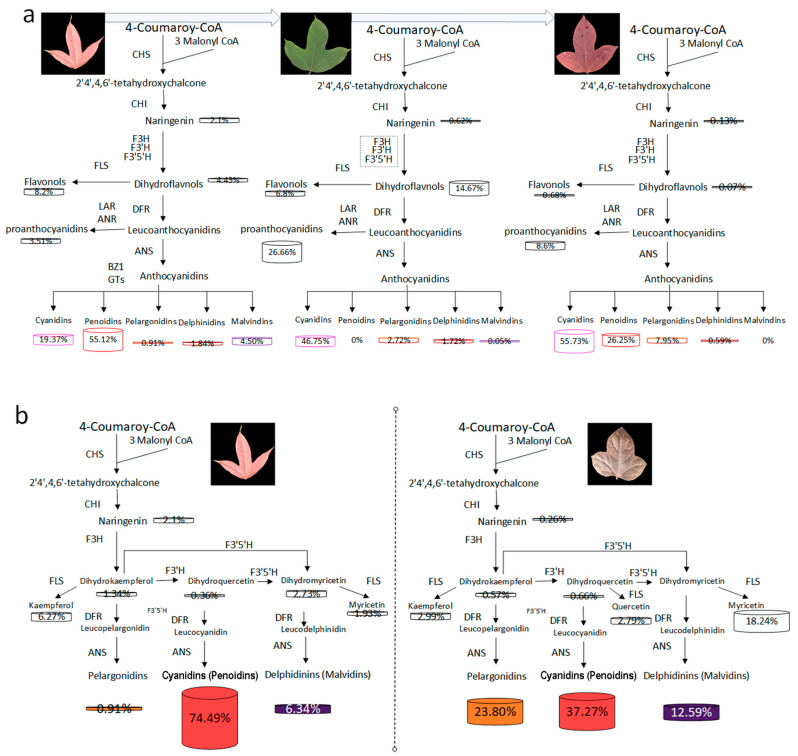
The models for anthocyanins biosynthesis in three season leaves of NLH (**a**), and processing in spring leaves of NLH and ZF (**b**), respectively. The abbreviations of the enzymes can be found in Supplementary Table S5.

Based on the qRT-PCR reactions, the expression levels of *LfCHS*, *LfCHI*, *LfF3H*, *LfF3′5′H*, *LfDFR*, *LfANS*, *LfBZ1*, *LfGT2*, and *Lf3MAT1* genes were significantly increased in NLHC compared to NLHX (Figure 4). These genes might be key factors due to the accumulation of anthocyanins in spring red leaves of *L. formosana*. Firstly, the *LfF3H*, *LfF3′H*, and *LfF3′5′H* genes can promote the branch of anthocyanins metabolism. Compared to NLHX, *LfF3H* and *LfF3′5′H* expressed higher levels in NLHC and the concentration of dihydrokaempferol in NLHC was 19 times in NLHX (Supplementary Table S1 and S2). Secondly, the *LfDFR* and *LfANS* genes also played an important role in anthocyanins biosynthesis. The concentrations of pelargoinidins and delphinidins were not significantly accumulated in NLHC, and kaempferol and myricetin were significantly accumulated, and none of quercetin was detected. Cyanidins and peonidins were found to be the main compounds in the red leaves of NLH. This indicates that the *LfDFR* and *LfANS* genes mainly catalyze the branched synthesis of dihydroquercetin into cyanidins and peonidins with higher expression levels in NLHC. Thirdly, after glycosyltransferase’s action, anthocyanidins would be transformed into stable anthocyanins as a final product (Figure 6a). *LfBZ1*, *LfGT2*, and *Lf3MAT1* were significantly up-regulated in the red leaves. Therefore, they might be key factors that contribute to the accumulation of cyandins and peonidins in the red leaves.

On the contrary, these genes were down-regulated in NLHX, which indicates that they might restrict the accumulation of anthocyanins in the green leaves. The *LfCHS* and *LfCHI* genes were upstream of the anthocyanin metabolic pathway, and they were nearly twice what was found in NLHC than NLHX. The competitive processing of *LfFLS* and *LfDFR* genes might reveal that fewer anthocyanins accumulated in NLHX. The *LfFLS* gene catalyzes the formation of flavonols from dihydroflavonols, which expressed higher levels in NLHX. This indicates that the anthocyanin biosynthesis was less active and flavonols were more active in summer. The concentration of flavonols was up to 20% of the detected flavonoids in NLHX, and the value was higher than in other seasons. The result verifies the assumption of low anthocyanidins biosynthesis activity. Although anthocyanidins were nearly 50% in the detected flavonoids in green leaves, the levels were far lower in other seasons. The presence of other flavonoids and chlorophylls could explain the colors of these anthocyanidins (Figure 6a).

Most of the upstream structure genes of anthocyanins biosynthesis pathway were more up-regulated in autumn than in the other seasons, especially for the expression level of *LfCHS* in NLHQ, which was 31.8 times that in NLHX and 14.5 times that in NLHC. The expression level of *LfCHI* in NLHQ was 52.9 times that in NLHX and 3.7 times that in NLHC. However, the expression levels of *LfF3H*, *LfF3′5′H*, *LfDFR*, *LfANS*, and *LfBZ1* in NLHQ were not significantly higher than those in NLHC. Meanwhile, in the detected secondary metabolites, cyanidins, pelargonidins, and procyanindin B showed the highest concentrations in dark red leaves compared to the leaves in other seasons. The concentration of pelargonidins in dark-red leaves was nearly six times higher than red leaves.

### 3.6. Candidates Responsible for Purple-Red Color in Spring Leaves of ZF

The *LfF3′5′H*, *LfDFR*, *LfANS*, *Lf5AT*, and *Lf5MAT2* genes showed a higher level in the variety of ZF, which might synergistically promote the accumulation of purple-red color in ZF leaves (Figure 4). ZFC showed lower expression levels in the genes of *LfCHS*, *LfCHI*, and *LfF3H* than NLHC. It might indicate ZFC had lower flavonoids compounds than NLHC. However, the concentration of anthocyanidins in ZFC showed a higher value than NLHC (Figure 2). The expression level of *LfF’3′5H* gene in ZFC was 2.4 times that in NLHC. The *LfANS* gene was 2.9 times as expressed in ZFC as in NLHC. In anthocyanidin, the biosynthesis pathway was more active in ZFC (Figure 6b). The *LfANR* and *LfLAR* genes expressed significantly lower levels in ZFC.

## 4. Discussion

### 4.1. Leaf Coloration among Different Seasons of NLH

NLH displayed variable colors in different seasons. The genes in the upstream structure of the anthocyanidins biosynthesis pathway were up-regulated in spring, down-regulated in summer, and then up-regulated again in autumn. Anthocyanins in NLHC and NLHQ were mainly cyanidin and peonidin derivatives, thus explaining the red and dark-red leaves. The anthocyanins were down-regulated in NLHX, and chlorophyll plays a dominant role in leaf color expression in summer. However, the different leaf colors in NLHX (red) and (NLHQ) dark-red might be influenced by several factors. Firstly, peonidins were the main pigment compounds in red leaves, whose concentration was 2.84 times, 60.83 times, and 29.96 times higher than cyanidin, pelargonidin, and delphinidin concentration. The peonidin synthesis pathway was dominant in the anthocyanins’ pathway, and its concentration accounted for 55.12% of the detected flavonoids, which in spring leaves was 3.15 times higher than in autumn leaves (Figure 6a). However, no upstream gene was significantly expressed higher in NLHC than NLHQ. *Lf3MAT1* genes showed the highest expression level in NLHC and might restrict the accumulation of peonidins in NLHQ. Although cyanidins and peonidins are both anthocyanin derivatives, they could change the colors in leaves from red to purple with acetylated sugars attached at different positions [26,27]. Secondly, the concentration of cyanidins in autumn leaves was two times, seven times, and 94.57 times higher than peonidins, pelargonidins, and delphinidins, and it accounted for 55.7% of detected flavonoids, whereas cyanidins accounted for 19.37% of detected flavonoids in spring leaves. Peonidins were higher accumulated in spring leaves and less in autumn, as well as the concentration of anthocyanidins. It indicated that anthocyanin biosynthesis was in a more dominant position of energy consumption competition in spring than it was in autumn. Although *LfCHS* and *LfCHI* were significantly up-regulated in autumn, the anthocyanidin biosynthesis pathway might not be the primary choice for energy consumption in NLH leaves. The *LfFLS* might limit the biomass of anthocyanins derivatives with increased up-regulation from spring to autumn. The amount of biomass that was generally turned into colorless flavonols and proanthocyanidins [12]. Thirdly, the concentration of pelargonidins in dark-red leaves was 5.84 times that in red leaves. The concentration of pelargonidins produced in the dark-red leaves accounted for 7.95% of the detected flavonoids, while the number of pelargonidins in red leaves accounted for 0.91%. Meanwhile, the expression of the *LfGT2* gene was up-regulated in dark-red leaves. Thus, pelargonidins (orange to brick-red, [28]) were also involved in the leaf coloration of NLHQ and might be connected with *LfGT2*.

### 4.2. Leaf Coloration between Two Varieties in Spring

The two varieties showed spring leaf color in red and purple-red. There were some differences in gene expression levels, as well as the composition of anthocyanidin metabolites. Some of the upstream genes (*LfCHS*, *LfCHI*, *F3H*, *F3′H*) showed higher expression levels in NLHC, but *LfDFR* and *LfANS* showed higher levels in ZFC with 2.7 times higher levels of total anthocyanidins concentration. The *LfF3′5′H* gene was up-regulated in ZFC, leading to accumulation of pelargonidins and delphinidins, which accounted for 36.39% of detected flavonoid concentration and was similar to the amount of cyanidins and peonidins (37.27%). The peonidins were stable at high pH in blue flowers [29] and were found in the red fruits at lower pH [30]. The concentrations of peonidins were similar in NLHC and ZFC. Thus, it might not be the reason for the purple-red leaves. Pelargonidins appeared as orange or red-colored pigments in some leaves and fruits [31,32,33]. The concentration of cyanidins in ZFC was 2.7 times higher than that in NLHC. Cyanidins are the major reddish-purple pigment in red-colored vegetables and purple corn [34]. The delphinidins were reported as a purple-colored pigment, and they could produce a blue color in flowers with higher pH conditions [35]. In this study, the two varieties were cultivated in the same soil with pH conditions around 5.5~6. The cyanidins were taken mainly as a part of the detected flavonoid concentration in NLHQ leaves, and its leaves did not present a purple color. Thus, the components of delphinidins might be the main contributor to purple-red color in ZF leaves. Co-pigmentation helped to stabilize the color of the leaves and flowers of the plant [36,37]. Co-pigmentation by delphinidins, pelargonidins, peonidins, and cyanidins might be the reason that the purple-red color was stable through the growing season in ZF, caused by the proportion of each of these anthocyanidins accounting for about 20 percent. Compared to the red leaves of NLHC, the proportion of myricetin might be one of the factors for the dark-red pigment in ZFC. The composition of anthocyanidin was the main reason for different leaf colors in *L. formosana*. The accumulation of delphinidins was significantly abundant in ZFC (Supplementary Table S1) as well as myricetins. Meanwhile, *LfF3′5′H* gene showed a higher expression level than *LfF3H* and *LfF3′H* genes. It indicated that *LfF3′5′H* gene had an effect on the accumulation of delphinidins and myricetins. Some key genes might regulate several branch metabolic pathways. However, it was complicated to assess the correlation between the metabolites and relative genes.

### 4.3. Mechanism of Leaves Coloration in L. formosana

The anthocyanidins’ derivatives might be beneficial for antioxidant, anti-photo oxidation, and antibacterial activities [38,39,40]. Although a series of research has been reported on flavonoid and anthocyanin metabolism in ornamental trees, the mechanism of *L. formosana* has not fully been addressed. The anthocyanin biosynthesis pathway is associated with TF complexes, which can regulate the structural genes [41,42]. The mechanism of different seasonal leaf colorations in NLH was speculated to be a large accumulation of peonidins and cyanidins turning the leaves red. Their ratio might cause different shades of red. The accumulation of pelargonidins caused the dark-red color of the leaves. The green leaves in summer were associated with only low amounts of anthocyanins, which were covered by chlorophyll. The expression levels of *LfF3′5′H*, *LfDFR*, and *LfBZ1* were higher in ZFX than NLHX, which might be the reason why the leaves of ZFX can stay purple-red in summer. To date, structural gene *LfDFR* and TF gene *LfMYB113* have been clarified to be related to anthocyanins in the senescence leaves [1], but *LfMYB113* was not significantly expressed in NLH and ZF leaves. In our study, twenty-four MYB DEGs were annotated and encoded by 45 unigenes in RNA-seq datasets among different seasons, and 16 MYB DEGs were up-regulated in red and dark-red leaves. However, only five TF genes (*LfMYBS3*, *LfMYBC*, *LfMYBS1*, *LfbHLH48,* and *LfbHLH63*) were significantly differentially expressed with seasonal change in NLH. Thus, these genes might be related to the leaf color variations among different seasons in NLH. MYBS1 was identified in rice [43], which was essential for mobilizing nutrient reserves in the endosperm to support seedling growth while receiving starving signals [44]. bHLH48 and bHLH63 were identified in Arabidopsis thaliana, which were responsible for binding activity. bHLH63 was a trigger for the flower to respond to blue light [45]. MYBC was a probable transcription factor that acts as a negative regulator of freezing tolerance in Arabidopsis [46]. This indicates that seasonal variation of leaf colors was influenced by multiple factors, such as temperature and light-period. The ultimate mechanism is probably complicated. The level of myricetin concentration accounted for 18.24% in ZFC, suggesting that the *LfDFR* and *LfFLS* were in the competition for their substrate dihydroflavonols. Some horticultural plant researchers also reported similar results [47,48]. The expression levels of *LfMYBC* were similar among the three seasons in ZF. Compared to the different expression in NLH, *LfMYBC* might have cooperated with *LfF3′5′H*, *LfDFR*, and *LfBZ1* for ZF to maintain its purple color in summer. Moreover, most pigmentation-related genes were significantly up-regulated in autumn leaves as well as in spring leaves. This indicates that they were not associated with cellular senescence in the leaves. Hence, further studies are needed to understand the gene network of red and purple leaf pigmentation pathways. Several TF genes in various TF families, such as NAC, ARF, and WRKY, are differentially expressed among differently colored leaves. Those TFs were also reported to have a connection with anthocyanidin accumulation. The identification of anthocyanin-related TFs might strengthen our thesis that color variation in *L. formosana* is a complicated regulatory mechanism. The mechanism would be clarified by examining correlation with transcription factors, miRNAs and knock down the suspected genes in the leaves of NLH or ZF in future work.

## 5. Conclusions

Anthocyanidin biosynthesis’s regulatory mechanisms were explored in the leaves of *L. formosna* in different seasons at the gene and metabolite levels. We screened out a subset of candidate genes related to leaf coloration, including 48 genes and 172 transcription factors that were associated with leaf colors. The accumulation of pelargonidins and delphinidins is the main factor in the difference between purple-red and red leaves. The reason for the seasonal colored leaves was the different concentrations of anthocyanins in NLH. On the contrary, the balance proportion of different kinds of anthocyanins was the main reason for constantly purple leaves in ZF. The anthocyanins were found in spring and summer leaves, and related genes were active at the same time. Thus, leaf coloration might not be associated with cellular senescence in ZF and NLH, which might be influenced by temperature and light. These results make an objective contribution to the colored leaf studies because it provides new insights for further study on complex physiological processes, biosynthesis mechanisms, and regulation of anthocyanidins in the leaves of *L. formosana*.

## Data Availability

Not applicable.

## Supplementary Materials

The following are available online at https://www.mdpi.com/article/10.3390/cimb44010018/s1.
