# Supplementary material for: Accumulation of Anthocyanidins Determines Leaf Color of Liquidambar Formosana as Revealed by Transcriptome Sequencing and Metabolism Analysis
Source: Curr Issues Mol Biol. 2022 Jan 7;44(1):242–56. doi: 10.3390/cimb44010018 (PMC8928986; doi:10.3390/cimb44010018)

Supplementary Figure S1: Pearson's distance correlation matrix of gene expression among twelve samples

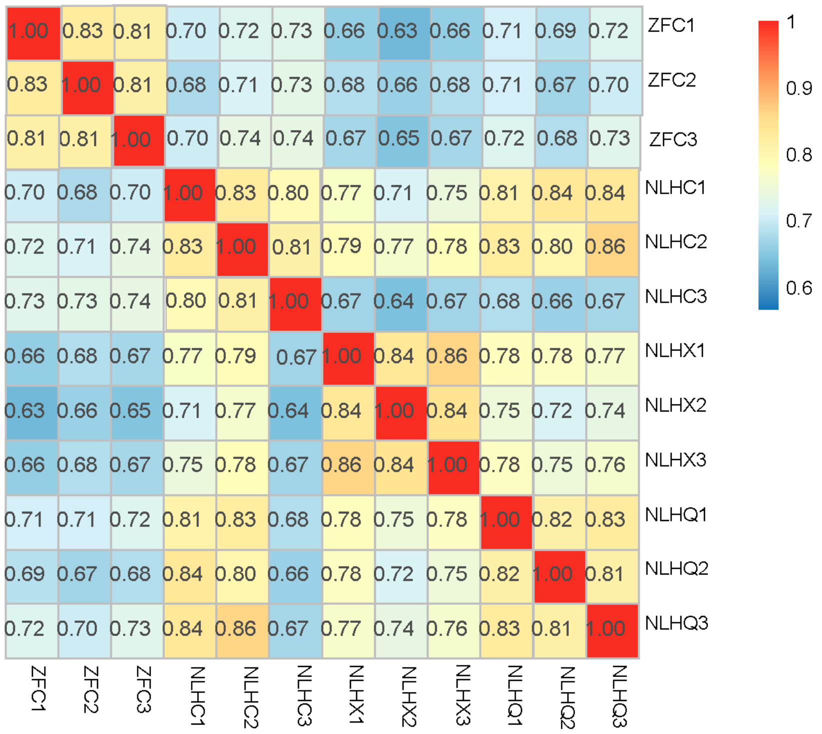

Supplement: Supplementary file 1 [file cimb-44-00018-s001.zip › Supplementary files/Supplementary Figure S1.pdf]
