# Supplementary material for: Accumulation of Anthocyanidins Determines Leaf Color of Liquidambar Formosana as Revealed by Transcriptome Sequencing and Metabolism Analysis
Source: Curr Issues Mol Biol. 2022 Jan 7;44(1):242–56. doi: 10.3390/cimb44010018 (PMC8928986; doi:10.3390/cimb44010018)
Supplement: Supplementary file 1 [file cimb-44-00018-s001.zip › Supplementary files/Supplementary Figure S4.pdf]

Supplementary Figure S4: Expression profile of leaf color related genes was Z-score normalized and hierarchically clustered in the heat map.

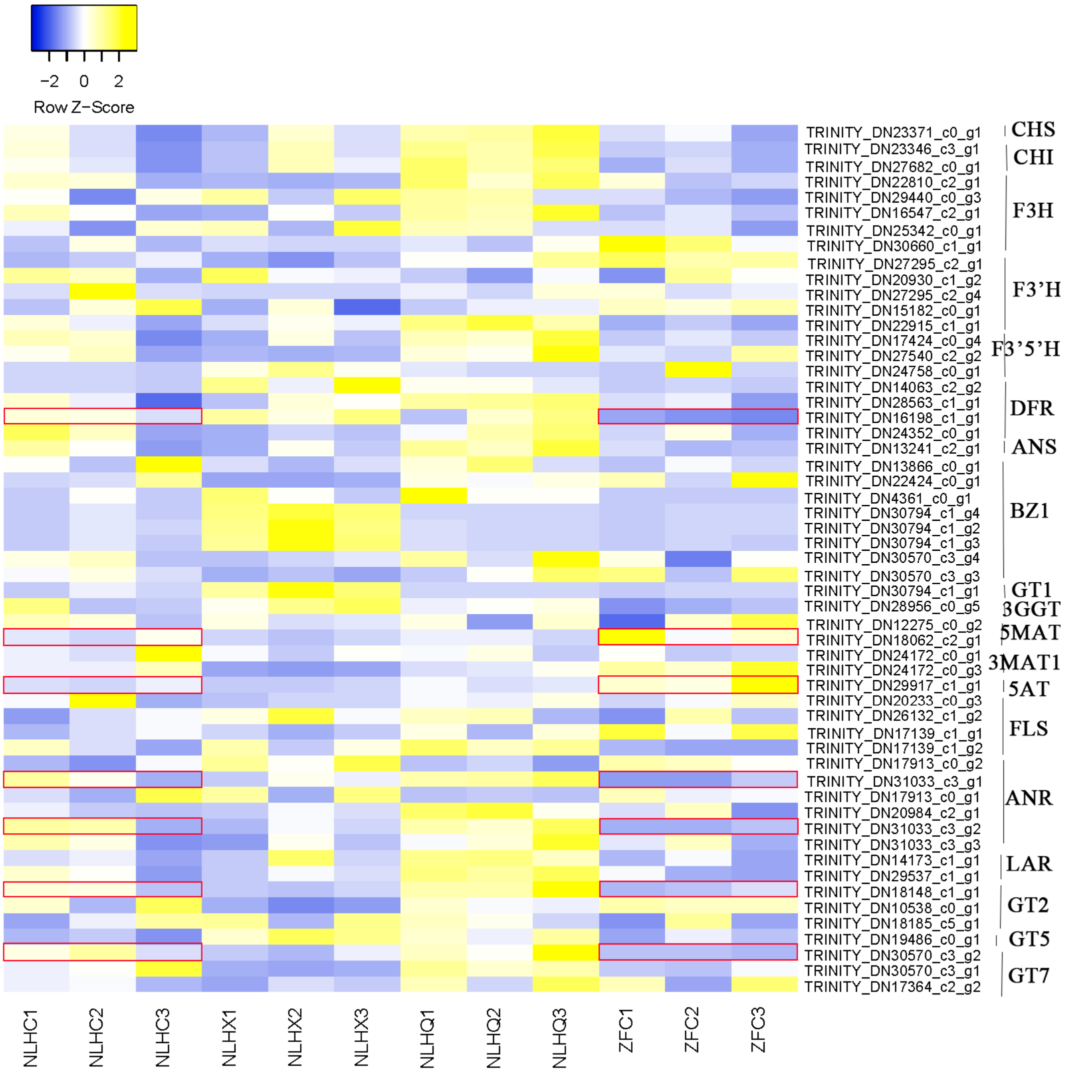

Notes: The DEGs between NLHC and ZFC were highlight in red box.
