# Supplementary material for: Accumulation of Anthocyanidins Determines Leaf Color of Liquidambar Formosana as Revealed by Transcriptome Sequencing and Metabolism Analysis
Source: Curr Issues Mol Biol. 2022 Jan 7;44(1):242–56. doi: 10.3390/cimb44010018 (PMC8928986; doi:10.3390/cimb44010018)
Supplement: Supplementary file 1 [file cimb-44-00018-s001.zip › Supplementary files/Supplementary Table S7.pdf]

Table S7 The primers used for qRT-PCR analysis

| Genes | Description | Sequences（5' - 3'） | Amplicons（bp） |
|---|---|---|---|
| *LfCHS* | Forward | CTCGCTTTACTGGATTGCCC | 229 |
| | Reverse | CCCAAAGAGTACACCCCACT | |
| *LfCHI* | Forward | AGCTCTCGACCTGAACACTC | 218 |
| | Reverse | TGGACCAATCTAACCCCACC | |
| *LfF3H* | Forward | CATGGGGCTAGAGAAGGAGG | 163 |
| | Reverse | ACCCACCTGATCTTGAAGCA | |
| *LfF3'H* | Forward | TCCTCACCACCACTTCCATC | 198 |
| | Reverse | TCATCTCTTCTCGGGCAAGG | |
| *LfF3'5'H* | Forward | GATCGAACGTGGGATGAAGC | 106 |
| | Reverse | ATCCGGTTTCCCACTACGTT | |
| *LfDFR* | Forward | CGGTTCCTGCAGATGATGTG | 193 |
| | Reverse | GAGGGAAGCTTTGATGACGC | |
| *LfANS* | Forward | GGAAGTTGGTGGCATGGAAG | 199 |
| | Reverse | TGAATTTGGGACGCACTTGG | |
| *LfBZ1* | Forward | TCCCGTCCCGAAATCCTAAC | 106 |
| | Reverse | ATCTTCCTTCCACTCTCGGC | |
| *LfGT1* | Forward | GGTGGCCTTTGTATGCTGAG | 133 |
| | Reverse | AACTCCCTCACTCGCTTCTC | |
| *LfGT2* | Forward | GCTCCCCAGTTACAAGTCCT | 166 |
| | Reverse | TTTCCCACACGTCCTCTACC | |
| *Lf5MAT2* | Forward | CGATCGAGTTACTTGTGCTGG | 134 |
| | Reverse | GATGGTGATGGGATAGAGGCA | |
| *Lf5AT* | Forward | AACTGAATCCCCATCCACGT | 137 |
| | Reverse | CGCAGACCACAATTCCTAGC | |
| *LfFLS* | Forward | GTCACCTGCAACACCAAGAG | 162 |
| | Reverse | CGATTGTAATGCCGAGGGTG | |
| *LfANR* | Forward | GTGGGTGGCTTCTGAGTAGTC | 186 |
| | Reverse | GATCAAGCCAGCAATCCAGG | |
| *LfLAR* | Forward | CTGGCTGCGAAATGGAATGA | 227 |
| | Reverse | TTCAACGACTTCATTGCCCG | |
| *LfMYBS3* | Forward | GCAACATGGCAGGTCATCAT | 208 |
| | Reverse | TGATGGCGTGAAGGTTGTTG | |
| *LfMYBS1* | Forward | AAGTACTTCTGGGCATGGCT | 116 |
| | Reverse | AGGTTGTTTCTCCTTGGCCT | |
| *LfMYB4* | Forward | TCTCCCCGCTATCAATGACC | 132 |
| | Reverse | AGTTGCAGGCTGAGATGGAT | |
| *LfMYBC* | Forward | TTTGTTCGGAGCAAGGCATC | 150 |
| | Reverse | CCCCGCATTAAACTCATCCG | |

| | | | |
|---|---|---|---|
| *LfbHLH63* | Forward | TGTTGCACTGTCTGAACACG | 209 |
| | Reverse | CTGAAGCTGCTGTCCTTGAC | |
| *LfbHLH48* | Forward | GGTGAAGAACGAACCGATGG | 146 |
| | Reverse | TGTTCTTCTTGCTCGGTCCT | |
| *LfbHLH67* | Forward | AGAAGCCATGGGAGAGGAAC | 188 |
| | Reverse | TTGTTCTTGGTTGCACTCGG | |
